# Association and interaction analysis of *PPARGC1A* and serum uric acid on type 2 diabetes mellitus in Chinese Han population

**DOI:** 10.1186/1758-5996-6-107

**Published:** 2014-10-01

**Authors:** Hui-Hui Wu, Nai-Jia Liu, Zhen Yang, Xiao-Ming Tao, Yan-Ping Du, Xuan-Chun Wang, Bin Lu, Zhao-Yun Zhang, Ren-Ming Hu, Jie Wen

**Affiliations:** Department of Endocrinology and Metabolism, Huashan Hospital, Fudan University, NO. 12 Wulumuqi Mid Road, Building 0#, Jing’an District, Shanghai 200040 China; Department of Endocrinology and Metabolism, Jing’an District Center Hospital of Shanghai, Shanghai, 200040 China; Department of Endocrinology and Metabolism, Xin Hua Hospital, Shanghai Jiao Tong University, Shanghai, 200020 China; Department of Endocrinology and Metabolism, Hua Dong Hospital, Fudan University, Shanghai, 200040 China

**Keywords:** *PPARGC1A*, Serum uric acid, Interaction, Type 2 diabetes mellitus, Chinese Han population

## Abstract

**Background:**

Peroxisome proliferator-activated receptor gamma coactivator-1α (PPARGC1A/ PGC-1α) is a ligand-activated transcription factor belonging to the nuclear hormone receptor superfamily. The activity of PGC-1α or genetic variations in the gene encoding the enzyme may contribute to individual variations in mitochondrial function and insulin resistance or diabetes. The objective of this study was to assess the extent to which *PPARGC1A* (rs8192678) and serum uric acid (UA) and its interaction impact on T2DM susceptibility in Chinese Han population.

**Method:**

We conducted a study in a cohort that included 1166 T2DM patients and 1135 controls, and was genotyped for the presence of the PPARGC1A rs8192678 polymorphisms. Genotyping was performed by iPLEX technology. The association between rs8192678 or UA and T2DM was assessed by univariate and multivariate logistic regression (MLR) analysis controlling for confounders. The interaction between rs8192678 and UA for T2DM susceptibility was also assessed by MLR analysis.

**Results:**

The generalized linear regression analysis failed to show an association between the *PPARGC1A* rs8192678 polymorphisms and T2DM. Interestingly, the present study provided data suggesting that the minor A-allele of *PPARGC1A* (rs8192678) had a protective effect against T2DM in subjects with higher level of UA (OR_int_ =1.50 95% CI: 1.06-2.12 for allele and P = 0.02, OR_int_ =1.63 95% CI: 1.17-2.26 for genotype and P = 0.004).

**Conclusion:**

The combination of higher level of UA and *PPARGC1A* (rs8192678) was an independent predictor for T2DM.

## Introduction

Type 2 diabetes mellitus (T2DM) is a complex metabolic disorder characterized by hyperglycemia as a result of pancreatic beta cell dysfunction and insulin resistance [1]. There is evidence demonstrating that genetic determinants as well as environmental factors may play a role in the pathophysiology of T2DM. Identifying rare and common genetic variants contributing to risk of or protection from T2DM will help uncover the complex mechanisms underlying T2DM, and provide crucial implications for the development of personalized medicine for diabetes mellitus [2].

Peroxisome proliferator-activated receptor gamma coactivator-1α (PPARGC1A*/*PGC-1α) is a ligand-activated transcription factor belonging to the nuclear hormone receptor superfamily, named after its ability to bind chemicals known to induce peroxisome proliferation [3]. Numerous studies have identified an important role for *PPARGC1A* in gluconeogenesis and insulin sensitivity as well as the beta-oxidation of fatty acids in the liver [4]. Plasma fasting insulin has been linked to the chromosomal region where the *PPARGC1A* gene is located [5], which verify the hypothesis that the gene may be a functional and positional candidate for T2DM. Moreover, endothelial *PPARGC1A* has been found to repress endothelial migration, thus potently inhibit endothelial function and angiogenesis, which further contribute to multiple aspects of vascular dysfunction in diabetes [6]. Associations of *PPARGC1A* variants with a range of other metabolic traits, including glucose concentrations, dyslipidemia and obesity have been reported [7–10]. The *PPARGC1A* rs8192678 polymorphism encodes a missense amino acid change, however, the activity of PGC-1α or genetic variations in the gene may contribute to individual variations in mitochondrial function and insulin resistance or diabetes [11, 12]. Recently, a new window has opened on the possible associations of *PPARGC1A* (rs8192678) with several metabolic related traits [13]. Furthermore, *PPARGC1A* (rs8192678) has also been found to be associated with a higher risk of T2DM in Indian and East Asian populations [14, 15]. Uncovering the effect of *PPARGC1A* variant on the susceptibility of T2DM in Chinese population and how it functions within the regulatory network will deepen our understanding of the biological roles of *PPARGC1A*.

In the relevant network, cellular *PPARGC1A* is regulated by signaling inputs that increase the transcription of the *PPARGC1A* gene and activity of *PPARGC1A* protein [16]. Emerging evidence has been provided to illustrate that *PPARGC1A* transcription increases with exercise, cold exposure, fasting and electro acupuncture [17–19]. It is thereby feasible that environment stimuli which could irritate the transcription of *PPARGC1A* gene also participate in modulation of T2DM susceptibility.

Serum uric acid (UA), as the final oxidation product of purine catabolism, has been associated with various clinical conditions, such as diabetes mellitus (DM), atherosclerotic disease and abdominal obesity [20–23]. Significant interactions between UA and age, triglycerides, as well as metabolic syndrome have also been reported in numerous studies [24–26]. There are few studies available on the effect of the interaction between UA and genetic variants on diseases. The objective of this study was to clarify whether the rs8192678 polymorphism or UA is associated with T2DM in Chinese population and to determine whether rs8192678 interacts with UA to impact on T2DM risk.

## Materials and methods

### Study population

The study protocol was approved by the Ethics Committee of Huashan Hospital, Shanghai, China. We carried out a study in a random sample of Chinese individuals to evaluate the association between *PPARGC1A* (rs8192678) and T2DM, as well as to determine whether the variant interacts with UA to influence T2DM susceptibility. Participants enrolled in the cohort, including 1166 T2DM patients and 1135 controls, were of Southern Han Chinese ancestry residing in the Shanghai metropolitan area. T2DM patients registered in the analysis were recruited from the Endocrinology and Metabolism outpatient clinics at Fudan University Huashan Hospital in Shanghai. Written consent was obtained from all patients before the study.

### Measurement

The subjects were interviewed for the documentation of medical histories, medications and regular physical examinations. Systolic and diastolic blood pressure (BP) values were the means of two physician-obtained measurements on the left arm of the seated participant. Body mass index (BMI) was calculated as the weight in kilograms divided by the square of height in meters. All participants underwent a complete hematological examination in the fasting state. Plasma glucose was quantified by the glucose oxidase-peroxidase procedure. Serum total cholesterol (TC), triglyceride (TG), high-density lipoprotein (HDL) cholesterol, urea nitrogen (UN), uric acid (UA) and alanine transaminase (ALT) levels were measured by an enzymatic method with a chemical analyzer (Hitachi 7600-020, Tokyo, Japan). Low-density lipoprotein (LDL) cholesterol levels were calculated using the Friedewald formula. The day-to-day and inter-assay coefficients of variation at the central laboratory in our hospital for all analyses were between 1% and 3%.

### Definition

Hypertension (HT) was defined as blood pressure ≥ 140/90 mmHg or history of hypertension medication. The classification of serum uric acid (UA) was based on the Chinese criteria [24]: UA ≥ 420 umol/L. Diabetes was defined according to 1999 WHO criteria [27]: fasting plasma glucose ≥ 7.0 mmol/L, and/or 2-hour plasma glucose ≥ 11.1 mmol/L in oral glucose tolerance test (OGTT). Known subtypes of diabetes were excluded based on antibody measurements and inheritance. The non-diabetic unrelated individuals meeting the following criterions were identified as the control population: 1) no family history of diabetes; 2) ≥ 45 years of age; 3) normal glucose tolerance verified by OGTT. The clinical characteristics of participants are summarized in Table 1.

**Table 1:** **The clinical characteristics of subjects**

Variable	Diabetes	Control
N	1166	1135
Age	65.46 ± 10.56	59.09 ± 7.85
Sex (male/female)	456/710	352/783
Height	160.2 ± 8.64	161.09 ± 7.65
Weight	64.85 ± 10.71	62.78 ± 10.15
SBP	139.52 ± 19.92	126.44 ± 16.92
DBP	82.88 ± 10.98	80.52 ± 10.13
UN	6.08 ± 1.63	5.62 ± 1.37
UA	0.30 ± 0.08	0.31 ± 0.08
FPG	8.39 ± 3.03	5.22 ± 0.38
PPG	15.05 ± 5.34	6.03 ± 1.04
TC	5.43 ± 1.11	5.35 ± 1.00
TG	2.00 ± 1.46	1.47 ± 1.06
HDL	1.29 ± 0.34	1.43 ± 0.36
LDL	3.11 ± 0.86	3.10 ± 0.79
ALT	28.44 ± 16.08	24.04 ± 13.69
rs8192678 (A/G)	490/670	475/646

Note: SBP-systolic blood pressure, DBP-diastolic blood pressure, UN-urea nitrogen, UA-uric acid, FPG-fasting plasma glucose, PBG-postprandial blood glucose, TC-total cholesterol, TG-triglyceride, HDL-high density lipoprotein, LDL-low density lipoprotein, ALT-alanine transaminase.

### SNP genotyping

Peripheral venous blood samples were collected from all study subjects, and the genomic DNA was isolated from whole blood by proteinase K digestion followed by phenol–chloroform extraction. *PPARGC1A* (rs8192678) was genotyped using iPLEX (Sequenom, San Diego, CA, USA) and detected by matrix-assisted laser desorption/ionization-time of flight mass spectrometry in a total of 2301 Chinese Han individuals. The genotype frequency was in Hardy-Weinberg equilibrium (P > 0.05) and there was a 99.9% genotype concordance rate when duplicated samples were compared across plates.

### Statistical analysis

The Kolmogorov-Smirnov test was used to determine whether continuous variables followed a normal distribution. Variables that were not normally distributed were log-transformed to approximate normal distribution for analysis. Results are described as mean ± SD or median unless stated otherwise. Differences in variables between T2DM and control were determined by unpaired *t*-test. Between groups differences in properties were accessed by *χ*^2^ analysis. Univariate logistic regression was performed to determine variables associated with T2DM and to estimate confounding factors possibly disturbing the relation of UA and/or *PPARGC1A* to T2DM. Multivariable logistic regression (MLR) was carried out to control potential confounders for determining independent contribution of variables to T2DM. For interaction analysis, MLR was conducted to include two main variables and its interaction item to determine the interaction effect. In order to better investigate the interaction between UA and *PPARGC1A* on T2DM, we performed two analyses according to alleles and genotypes of *PPARGC1A* that were present in the study population by additive model. Odds ratios (OR) with 95% confidence intervals (CI) were calculated as measures of association of UA and/or *PPARGC1A* with T2DM. Results were analyzed using the Statistical Package for Social Sciences for Windows version 16.0 (SPSS, Chicago, IL, USA). Tests were two-sided and a p-value of < 0.05 was considered significant.

## Results

### Clinical characteristics of subjects

The baseline clinical characteristics of the 2301 subjects were listed in Table 1. There are 456 males and 710 females (mean age, 65.46 ± 10.56 years) in cases and 352 males and 783 females (mean age, 59.09 ± 7.85 years) in controls. Diabetic patients had more weight than controls. SBP, DBP, FPG, PPG, TC and TG levels were higher in cases than those of controls, while HDL level was lower in cases. Serum UA, ALT and LDL levels were similar between the two groups. The minor allele (A) frequency of *PPARGC1A* was 42.24% and 42.37% in cases and controls, respectively.

### Univariate and multiple logistic regression analysis for diabetes

To estimate the association of various clinical factors and T2DM, univariate logistic regression models were developed to include age, sex, hypertension, lipid profiles, UA and the rs8192678 polymorphisms (Table 2). The univariate logistic analyses indicated that age, sex, BMI, hypertension and TC were significantly associated with T2DM (P < 0.05 for all), however, there were no significant association of UA and SNP (rs8192678) with T2DM (P = 0.744 and 0.928, respectively). The proportion of T2DM was 52.17% and 51.75% in low UA group and high UA group, respectively, but the difference was not statistically significant between the two groups (P = 0.744).

**Table 2:** **Univariate logistic regression analysis for diabetes**

Variable	***β***	S.E.	P Value	OR	95% CI
Age	0.07	0.003	<0.001	1.07	1.07-1.08
Sex	−0.35	0.06	<0.001	0.70	0.62-0.80
BMI	0.59	0.08	<0.001	1.81	1.54-2.14
HT	1.21	0.09	<0.001	3.36	2.83-3.99
TC	0.07	0.03	0.01	1.07	1.02-1.14
TG	0.46	0.03	<0.001	1.59	1.48-1.70
UN	0.21	0.02	<0.001	1.24	1.19-1.29
UA	0.03	0.09	0.74	1.03	0.87-1.22
*PPARGC1A*	−0.005	0.06	0.93	0.99	0.88-1.12

Note: *PPARGC1A* with SNP (rs8192678), BMI- body mass index, TC-total cholesterol, TG-triglyceride, UN-urea nitrogen, UA-uric acid, HT-Hypertension.

### UA by *PPARGC1A*interaction analysis for diabetes

**Figure 1:**
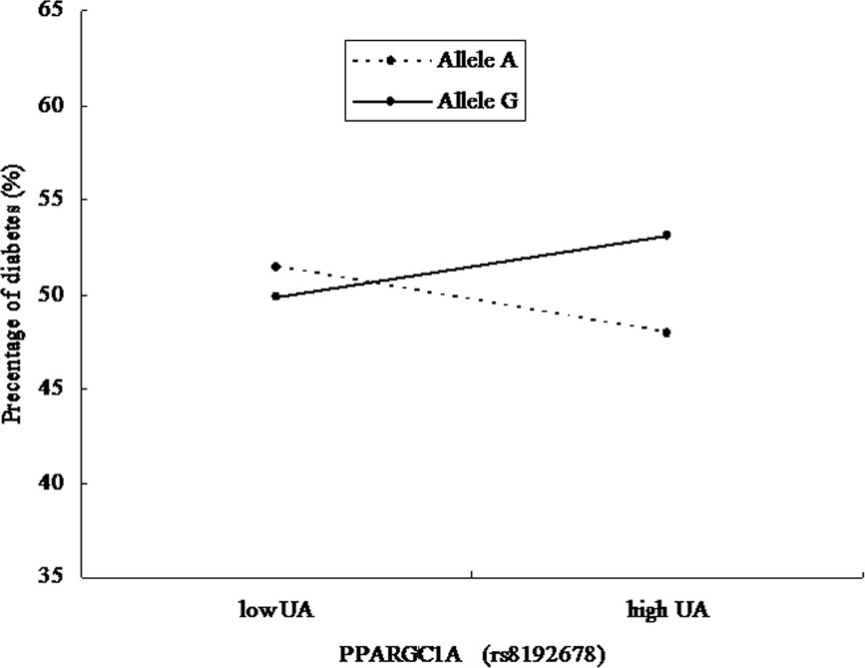
**The interaction effect analysis of UA and** ***PPARGC1A*** **for diabetes.**

The interaction item between UA and *PPARGC1A*, as well as the interaction effect were detected in MLR models after adjustment for relevant potential confounders, showing a certain relationship between them (P = 0.02, OR_Int_ = 1.50, 95% CI 1.06-2.12, shown in Table 3 and Figure 1). In model of rs8192678 with allele variable, MLR models were developed to include the two main effect variables: UA and rs8192678. In another model of rs8192678 with genotype variable, the interaction term of UA and rs8192678 was detected by the same method (P = 0.004, Table 4), as well as the interaction effect (OR_Int_ = 1.625, 95% CI 1.172-2.255). In these two models, MLR models signified that UA or rs8192678 alone was not significantly associated with T2DM. The interaction term of UA and SNP rs8192678 which was detected in the two models (allele and genotype models) only confirmed a significant association between this SNP and T2DM in high UA group (P = 0.018 for allele analysis and P < 0.01 for genotype analysis, data not shown).

**Table 3:** **The interaction effect analysis of UA and allele of** ***PPARGC1A*** **(rs8192678) for diabetes**

Variable	***β***	S.E.	***P***Value	OR	95.0% C.I
UA	−0.12	0.11	0.28	0.89	0.71-1.10
rs8192678(A/G)	−0.06	0.06	0.32	0.94	0.83-1.06
UA by rs8192678(A/G)	0.41	0.18	0.02	1.50	1.06-2.12

Note: Variable - UA, *PPARGC1A* (rs8192678), UA by *PPARGC1A* (rs8192678); Adjusted for Age, Sex, HT, BMI.

**Table 4:** **The interaction effect analysis of UA and genotype of** ***PPARGC1A*** **(rs8192678) for diabetes**

Variable	***β***	S.E.	***P***Value	OR	95.0% C.I
UA	−0.19	0.19	0.33	0.83	0.57-1.20
rs8192678(AA/AG/GG)	−0.04	0.07	0.60	0.96	0.84-1.10
UA by rs8192678	0.49	0.17	0.004	1.63	1.17-2.26

Note: Variable - UA, *PPARGC1A* (rs8192678), UA by *PPARGC1A* (rs8192678); Adjusted for Age, Sex, HT, BMI.

## Discussion

Our current study evaluated the association between *PPARGC1A* and T2DM in a case-control trial, subjects enrolled in which were of Chinese Han population including 1166 T2DM patients and 1135 controls. Genotyping was performed for *PPARGC1A* (rs8192678) in the cohort. This is the first report to our knowledge of an interaction analysis on T2DM susceptibility based on variables of UA and *PPARGC1A*.

Defect in mitochondrial oxidative phosphorylation have been linked to insulin resistance [28], and there is also evidence suggesting that polymorphisms in PPARGC1A are associated with an increased relative risk of type 2 diabetes, defects in insulin secretion, and lipid oxidation [29]. PPARGC1A is a transcriptional regulator of genes responsible for mitochondrial biogenesis and fat oxidation. Consistently, *PPARGC1A* (rs8192678) was reported to be associated with T2DM in a European population, as well as diabetic nephropathy (DN) in an Asian Indian population [30, 31]. These additional findings, which so far have been observed in numerous studies, showed that *PPARGC1A* may be a potential genetic marker for T2DM. In our present study, the interaction of rs8192678 (G allele) and high concentration of UA conferred a high risk of T2DM (OR in Tables 3 and 4, Figure 1), while a combination of the A-allele and high UA concentration seemed to protect against T2DM. However, there is little evidence to demonstrate *PPARGC1A* (rs8192678) to be an independent risk factor of T2DM. Such results may be attributed to the limited number of participants which had insufficient statistical power to detect a slight effect of the common polymorphism in *PPARGC1A* on T2DM susceptibility. A larger sample size, therefore, is necessary to detect the association between this *PPARGC1A* genetic variant and T2DM. In addition, another possibility of this puzzling phenomenon is the diverse ethnic/regional backgrounds, that is, findings might vary by population because of the underlying unobserved genetic variation.

As mentioned above, the *PPARGC1A* gene, transcription of which increases with environment stimuli, is a master regulator of mitochondrial genes. Interestingly, recently studies showed an improved intrinsic mitochondrial function in *PPARGC1A*-overexpressing mice, but only when fatty acids are used as a substrate [32]. Associations between *PPARGC1A* genotypes and alcohol consumption have been observed in a Mediterranean population [33]. The persuasive arguments confirm the hypothesis that intake of alcohol and fatty acid infusion/supplementation may influence T2DM risk through their modulation in *PPARGC1A* transcription. On this basis, we suspected that UA may also modify the transcription of *PPARGC1A* which further regulate the morbidity of diabetes mellitus. Our study is the first analysis to detect the interaction effect of UA and *PPARGC1A* (rs8192678) on T2DM susceptibility. It is noteworthy that the risk imparted by the wild G-allele was statistically significantly higher than that of A-allele in subjects with higher levels of UA (P < 0.05 for allele analysis and P < 0.01 for genotype analysis), despite the lack of a direct link between UA level and T2DM. The amusing results prompted the speculation of possible interaction between *PPARGC1A* (rs8192678) and UA level in determining overall T2DM risk. However, the underlying mechanism is yet to be determined.

Several limitations of the study warrant comment. First, subjects participated in the study were recruited from Shanghai, so they may not have been representative of China as a whole. Second, since our study was performed on Chinese individuals, these findings may not be relevant to people of other ethnicities. Also, it is important to confirm the verdict with additional investigations using a larger sample to systematically evaluate the likely interaction effect of *PPARGC1A* variants and UA on T2DM risk.

In conclusion, the possible association of *PPARGC1A* (rs8192678) with T2DM has not been confirmed in the present study. Interestingly, the data also suggested that the minor A-allele of *PPARGC1A* (rs8192678) had a protective effect against T2DM in subjects with higher levels of UA. However, a functional study, such as gene-targeting in mice, is needed to clarify the role of *PPARGC1A* as a whole.
